# Supplementary material for: Quantitative Multiparametric Ultrasound (mpUS) in the Assessment of Inconclusive Cervical Lymph Nodes
Source: Cancers (Basel). 2022 Mar 22;14(7):1597. doi: 10.3390/cancers14071597 (PMC8997164; doi:10.3390/cancers14071597)
Supplement: Supplementary file 1 [file cancers-14-01597-s001.zip › cancers-1618390-supplementary.pdf]

cancers

MDPI

Article

Quantitative Multiparametric Ultrasound (mpUS) in the As-Sessment of Inconclusive Cervical Lymph Nodes

Markus H. Lerchbaumer, Katharina Margherita Wakonig, Philipp Arens, Steffen Dommerich and Thomas Fischer

Supplementary Material.

Table S1. Contrast-enhanced ultrasound analysis of general cohort. All metric variables are given as median with corresponding standard deviation. Abbreviations: TTP denotes time to peak; mTTI, transit time; PE, peak enhancement; RT, rise time; WiAUC, wash-in area under the curve; WiR, wash-in rate; WiPi, wash-in perfusion index; WoAUC, wash-out area under the curve; WiWoAUC, wash-in/wash-out area under the curve; FT, fall time; WoR, wash-out rate.

**Contrast-Enhanced Ultrasound (*n* = 98)**			
	Benign (*n* = 34)	Malignant (*n* = 64)	*p*-Value
**Lin**	116445.85 ± 353924.19	123339.71 ± 404779.02	> 0.05
**TTP (s)**	8.18 ± 2.71	8.52 ± 2.59	> 0.05
**mTTI (s)**	38.8 ± 28.65	52.05 ± 63.13	> 0.05
**PE**	959188.62 ± 3076141.37	1132352.14 ± 3974686.15	> 0.05
**RT (s)**	5.37 ± 1.78	5.85 ± 2.19	> 0.05
**WiAUC**	2240176.88 ± 6730820.83	3063429.61 ± 11173425.6	> 0.05
**WiR**	372230.53 ± 1251089.89	372547.11 ± 1265149.67	> 0.05
**WiPI**	601649.24 ± 1929603.57	704199.7 ± 2455057.61	> 0.05
**WoAUC**	3862267.84 ± 11482342.4	4802503.87 ± 16689799.6	> 0.05
**WiWoAUC**	6102444.61 ± 18194135.2	7865933.4 ± 27746921.3	> 0.05
**FT (s)**	11.44 ± 4.60	14.36 ± 8.6	> 0.05
**WoR**	173184.49 ± 571251.16	200415.06 ± 735514.91	> 0.05

Table S2. Contrast-enhanced ultrasound analysis of subgroup “Solbiati-Index > 2“. All metric variables are given as median with corresponding standard deviation. Abbreviations: TTP denotes time to peak; mTTI, transit time; PE, peak enhancement; RT, rise time; WiAUC, wash-in area under the curve; WiR, wash-in rate; WiPi, wash-in perfusion index; WoAUC, wash-out area under the curve; WiWoAUC, wash-in/wash-out area under the curve; FT, fall time; WoR, wash-out rate.

**Contrast-Enhanced Ultrasound (*n* = 25)**			
	Benign (*n* = 16)	Malignant (*n* = 9)	*p*-Value
**Lin**	208874.77 ± 491519.34	9996.06 ± 29071.5	> 0.05
**TTP (s)**	8.25 ± 2.56	8.93 ± 1.8	> 0.05
**mTTI (s)**	34.57 ± 26.41	42.72 ± 22.01	> 0.05
**PE**	1892053.03 ± 4344410.95	58919.62 ± 173935.83	> 0.05
**RT (s)**	5.25 ± 1.67	5.88 ± 1.09	> 0.05
**WiAUC**	4134349.87 ± 9381491.06	181138.75 ± 532248.05	> 0.05
**WiR**	755992.07 ± 1771420.95	17112.39 ± 50637.85	> 0.05
**WiPI**	1184165.98 ± 2725209.69	37265.28 ± 109982.92	> 0.05
**WoAUC**	6811490.10 ± 15740943.3	321511.03 ± 941987.11	> 0.05
**WiWoAUC**	10945839.7 ± 25118069.6	502649.76 ± 1474234,74	> 0.05
**FT (s)**	10.7 ± 4.35	12.26 ± 2.98	> 0.05
**WoR**	355004.79 ± 355004.79	7148.72 ± 21196.03	> 0.05

Table S3. Contrast-enhanced ultrasound analysis of subgroup “Short axis diameter <1cm”. All metric variables are given as median with corresponding standard deviation. Abbreviations: TTP denotes time to peak; mTTI, transit time; PE, peak enhancement; RT, rise time; WiAUC, wash-in area under the curve; WiR, wash-in rate; WiPi, wash-in perfusion index; WoAUC, wash-out area under the curve; WiWoAUC, wash-in/wash-out area under the curve; FT, fall time; WoR, wash-out rate.

	**Contrast-Enhanced Ultrasound (*n* = 45)**		
	Benign (*n* = 27)	Malignant (*n* = 18)	*p*-Value
**meanLin**	123840.67 ± 387687.53	45911.75 ± 109515.07	> 0.05
**TTP (s)**	8.33 ± 2.82	7.91 ± 2.87	> 0.05
**mTTI (s)**	42.14 ± 30.4	32.77 ± 24.7	> 0.05
**PE**	1121399.16 ± 3433057.41	467533.02 ± 1212756.07	> 0.05
**RT (s)**	5.51 ± 1.81	5.35 ± 2.33	> 0.05
**WiAUC**	2450676.39 ± 7420124.02	1075655.33 ± 2719378.16	> 0.05
**WiR**	448046.91 ± 1397707.25	177994.01 ± 470568.09	> 0.05
**WiPI**	701845.828 ± 2153151.89	290738.07 ± 754994.18	> 0.05
**WoAUC**	4037905.9 ± 12432549.6	1681654.71 ± 4276539.57	> 0.05
**WiWoAUC**	6488582.14 ± 19849431.8	2757310.06 ± 6992273.45	> 0.05
**FT (s)**	11.76 ± 4.54	12.24 ± 6.81	> 0.05
**WoR**	210390.36 ± 637763.45	98219.89 ± 257868.03	> 0.05
